# Spanish-Language News Consumption and Latino Reactions to COVID-19

**DOI:** 10.3390/ijerph18189629

**Published:** 2021-09-13

**Authors:** Barbara Gomez-Aguinaga, Ana L. Oaxaca, Matt A. Barreto, Gabriel R. Sanchez

**Affiliations:** 1 School of Public Adminisitration, University of Nebraska, Omaha, NE 68182, USA; 2 Department of Political Science, University of California, Los Angeles, CA 90095, USA; aoaxaca@ucla.edu (A.L.O.); barretom@ucla.edu (M.A.B.); 3 Department of Political Science, University of New Mexico, Albuquerque, NM 87131, USA; sanchezg@unm.edu

**Keywords:** COVID-19, ethnic media, Spanish-language news, pandemic, health information retrieval

## Abstract

While the literature on infectious disease outbreaks has examined the extent to which communication inequalities during public health emergencies exacerbate negative outcomes among disadvantaged individuals, the implications of ethnic media consumption among minority groups during these crises are underexplored. Making use of the first nationally representative survey of US Latinos (*N* = 1200) on the impact and reactions to COVID-19, this study examines the implications of Spanish-language news media consumption on source credibility and attitude formation during the COVID-19 pandemic among Latinos and immigrants from Latin America. Through a series of statistical analyses, this study finds that ethnic news consumption is strongly associated with trust in Spanish-language journalists, whereas mainstream media consumption is not associated with trust in English-language journalists. More importantly, this study finds that source credibility, particularly in Spanish-language journalists, matters for Latinos as it is associated with more positive assessments of state and local officials providing adequate information about COVID-19. This study illuminates the importance of non-traditional media among racial minorities, who account for almost 40% of the US population, and highlights the importance of shared backgrounds in source credibility among linguistically diverse groups in the United States during a public health crisis.

## 1. Introduction

As the COVID-19 pandemic continues to threaten the health and claim the lives of hundreds of thousands of Americans, studies have tried to examine the role of mass media and public health communications during the current public health emergency. Scholars argue that the news media has played a key role in disseminating valuable and lifesaving information about the effects of COVID-19 on public health, as well as steps to reduce the threat of infection [1,2]. However, there have been several inconsistent messages from the media, officials, and prominent leaders, contributing to mass misinformation and leading to public confusion and risk [3]. Policies and recommendations regarding masks, social distancing, non-essential business, and school closures have varied and evolved across media and all levels of government, creating inconsistent messages that have affected the American public. For underrepresented groups, in particular, these issues have the potential to create problems regarding access to reputable information and adverse health effects [4].

The extant literature on infectious disease outbreaks explores how communication inequalities during public health crises produce negative outcomes for individuals with lower socio-economic status, communication barriers, and lower knowledge levels [4,5,6]. In addition, lack of media trust can exacerbate the already deadly impact of a pandemic by causing members of the public to disregard appropriate steps that keep themselves and others safe [7]. These issues are crucial during the COVID-19 pandemic as inequalities in the United States continue to rise and declining levels of media trust among the American public persist [8]. 

Literature around trust and credibility, and its consequences on health behavior during pandemics, presents evidence that better knowledge and source credibility can induce behaviors that lead to better health outcomes [4]. Additionally, researchers have explored the role of language and trust in sources, finding that the use of minority languages can have important nuances for media trust among linguistic minorities such as Spanish-speaking Latinos [9,10,11,12]. This issue is vital during the COVID-19 pandemic as US Latinos and other racial minority groups are significantly more likely to be infected, hospitalized, and die from COVID-19 than non-Hispanic Whites [13].

While studies have found that access to news media can make a substantial difference in accessing factual information during public health emergencies [14], the role of ethnic and non-traditional media during infectious disease outbreaks like COVID-19 is empirically understudied. This is an important area of study in a growingly diverse country like the United States, where more than 60 million Americans regularly get news and other information from non-traditional media [15]. Spanish-language news media is a crucial source of information for US Latinos, who are the largest ethnic group in the country; most of them are proficient in Spanish, the largest spoken minority language in the country [16,17]. Hence, this study explores the relationship between Spanish-language news consumption, source credibility, and health attitudes among US Latinos during the COVID-19 pandemic. It specifically analyzes the extent to which Spanish-language news media consumption leads to greater trust in journalists and the extent to which source credibility impacts the attitude formation of Latinos during pandemics. Given that intra-group language differences are linked to variation in the consumption and trust of information among linguistic minorities [9,10,18], we argue that Latinos who engage at higher levels with Spanish-language news consumption display higher levels of trust in their sources and more positive assessments of COVID-19 information.

### 1.1. Media Use and Source Credibility 

Trust has been considered an important factor that influences news media consumption [18,19]. However, media trust has increasingly become an issue of concern as surveys and polls reveal that Americans’ trust in news is at historically low levels [8,20]. Studies show that decreasing levels of media trust result from multiple issues, including the changing nature of journalism, increasing availability of partisan or ideological news, and attacks on journalism and “fake news” from political leaders [21]. Furthermore, studies have found that media trust can also vary by demographic characteristics of the public, such as age [22], income [23], educational attainment [24], gender [25], and race, with non-Hispanic Whites having lower levels of media trust than other ethno-racial groups [26,27]. 

Communication studies divide the analysis of media trust into three different areas. Medium/channel credibility refers to the trust that people have in a platform in which a message is delivered, including newspapers, radio, television, and social media [21,28]. Message credibility deals with the content of messages regardless of the medium or platform in which they are presented [29]. Source credibility denotes trust in information providers—typically individual communicators such as reporters, news anchors, or public speakers [30,31]. In addition to providing a more comprehensive understanding of media trust, the three areas engage in the complex and multidimensional nature of the concept.

Of the three components of media trust, source credibility provides an opportunity to analyze the implications of the presence of underrepresented groups in news media during the COVID-19 pandemic. By emphasizing the characteristics of individual communicators, studies of source credibility explore the extent to which demographic characteristics of information providers, such as race and gender, impact media trust among news consumers. Regarding gender, scholars have found that male news consumers tend to have greater evaluations of content created by male reporters than content from female reporters [32,33,34]. Similarly, studies have found that shared racial backgrounds also impact source credibility, with individuals of the same racial group being more trustful of the information provided by co-ethnic messengers than information providers from different groups [35,36,37]. This occurs because perceived similarities, especially among underrepresented groups, can trigger subsequent thoughts, decisions, and evaluations, as the Social Comparison Theory (SCT) posits [38]. SCT contends that “we tend to compare ourselves to others, and the greater the similarity we perceive, the more likely we are to attend to and trust what [individual communicators] say” [39]. In other words, SCT suggests that demographic similarities such as race and ethnicity can be salient and influence consumers’ minds while evaluating messages.

One of the problems of US news media is the lack of diversity and inclusion of underrepresented groups. The US Census Bureau estimates that while racial and ethnic minorities account for about 40% of the US population, they account for less than 17% of the staff in print and online newsrooms [40]. This lack of diversity in newsrooms is salient for US Latinos, who account for only 5% of newsroom staff despite being the largest ethnic group in the country [41]. Nevertheless, the rise of new information technologies has benefited Latinos and other minority groups through the emergence of ethnic media, which are commonly defined as “broadcast, print, and digital communication” alternatives to mainstream media, designed to “serve a particular cultural or racial group” [42]. Most of this media originates within the US mainland and focuses on issues often ignored by mainstream news [41,43]. The availability and consumption of Spanish-language news media and ethnic media have significantly increased in the past decades, becoming powerful sources of information that reach about 75% of US Latinos and ethno-racial minorities in the country [15,43].

A core component that differentiates Spanish-language news media from mainstream media is the abundant presence of Latinos and the dissemination of information in a language other than English [44]. Not only does Spanish-language news reflect the experiences of Latinos in the United States, but its news anchors and reporters also mirror the linguistic and ethnic backgrounds of Latino consumers [45,46]. Given that studies have found that shared backgrounds can have an impact on perceptions of source credibility, particularly among underrepresented groups, and that Spanish-language news media mirrors the linguistic and ethnic background of Latino consumers, we expect that the consumption of Spanish-language news media is associated with higher levels of source credibility in Spanish-language journalists.

H_1_ (SL news consumption and source credibility): Latino consumers of Spanish-language news have greater levels of source credibility in Spanish-language journalists than English-language journalists.

### 1.2. Source Credibility and Health Behavior

Health attitudes and behavior during infectious disease outbreaks have been researched widely. Many studies have focused on understanding the social determinants of compliance for recommended behaviors during infectious disease outbreaks [47,48,49]. When investigating compliance with vaccines and guidelines during public health emergencies, researchers have found that fear of contracting the illness leads to refusal to comply with vaccinations and safety guidelines [49,50]. Nonetheless, news consumption and media trust can counteract such attitudes. Lee found that people receiving health information from trusted media believe it is more credible than information through interpersonal channels, arguing that the presence of experts on mass media made this relationship possible [50]. Additionally, studies have found that mass media can provide accessible health information and knowledge on preventative behaviors to wide audiences, particularly during public health emergencies [4,51,52]. Many studies conclude that providing racially and ethnically diverse communities with trusted messages can lead these groups to adhere to public health recommendations [53,54], which is of vital importance during public health emergencies that exacerbate health inequalities among racial and ethnic minorities [13,55].

The news media can take an important role in public health behavior amidst a world-wide health crisis. Studies have found that communication inequalities disproportionally affect people with lower socio-economic status as unreliable information can linger in these communities for longer periods of time [56]. However, media trust can be highly salient to dismantling misinformation and promoting compliance with recommended health behaviors, particularly among vulnerable groups [2,57]. While few studies have examined the differences in media trust among English- and Spanish-speaking Latinos [11], the implications of media trust and source credibility by type of news consumption (ethnic vs. mainstream news) have been understudied, especially during public health emergencies such as the COVID-19 pandemic. 

Given that literature on media trust finds that source credibility can have important implications on health behavior and attitudes during outbreaks and that intra-group language differences are important nuances for media trust [2,12,57], we expect that greater levels of trust in journalists is associated with positive reactions to actions taken by state and local officials during the COVID-19 pandemic.

*H_2_ (Source credibility and attitude formation*): Latinos with greater levels of trust in journalists are more likely to have more positive attitudes towards the actions that state and local officials are taking during the COVID-19 pandemic. 

## 2. Materials and Methods

To analyze the implications of SL news media consumption on source credibility and attitude formation during the COVID-19 outbreak, we make use of the SOMOS COVID-19 Crisis National Latino Survey, which was conducted from 7 to 12 April 2020 during a critical point in the US outbreak, when over 189,000 new COVID-19 cases and more than 12,000 deaths were reported [13,58]. The survey has a nationally representative sample of 1200 US Latinos with a margin of error of +/−2.8%. Latino adult participants were randomly recruited from a combination of landline and cell phones, as well as online panel vendors with large national Hispanic samples. Listed were de-duped to ensure each respondent only had one opportunity to be selected for an interview. Overall, the telephone portion had a response rate of 6.2% and a cooperation rate of 48.5%. The online portion had a response rate of 37.3% and a cooperation rate of 70.4%. The survey averaged 12 min online and 19 min over the telephone. Final data were compared to the 2018 Census ACS demographic profile of Latino adults, and post-stratification weights were included to balance gender, age, education, and geography so the resulting data matched the national demographic profile of Latino adults. Critical to our analysis, the survey was conducted in English (59%) and Spanish (41%), allowing for analyses across bilingual and Spanish-speaking Latinos [58]. 

To test H1, the dependent variable is trust in English or Spanish-language journalists. This was a split sample, in which half of the respondents were randomly assigned to the following question: “On a scale of 0 to 10, where 0 means “do not trust at all” and 10 means “completely trust”, how much do you trust [English-language news journalists/Spanish-language news journalists] to provide accurate information and helpful advice related to the coronavirus outbreak?” This design sample provides an opportunity to test H1. The main independent variables to test H1 are consumption of English and Spanish-language news media; see Appendix A for the exact wording of these variables, which were measured independently through two separate questions. 

The rest of the analyses makes use of three dependent variables to test H2. The first is collective health, which measures whether respondents prefer (0) reopening the economy or favor (1) government intervention to prevent the spread of COVID-19. The second and third dependent variables testing H2 are related to the effectiveness of information from state and local officials about COVID-19, including stopping the spread of COVID-19 and the location of the nearest testing site; responses for these questions ranged from (1) “strongly disagree” to (4) “strongly agree”. Half of the survey respondents were randomly assigned one of the two questions, consequently yielding a smaller number of observations. See Appendix A for the exact wording of the variables, their distribution, and sample size. 

The analyses testing H2 have controlled for news consumption specifically to obtain information about COVID-19 and public risk perception of COVID-19 as literature in health communication has found that these variables can impact health behavior during public health emergencies [59,60,61]. Additional control variables in our analyses include age, education, gender, nativity, partisanship, language of survey, and Cuban ancestry as existing literature has identified them as influential in the public attitudes among Latinos and ethno-racial minorities [10,62,63]. Table A1 in Appendix A presents a comprehensive list of the wording and summary statistics of all the covariates used in this study. 

As the analyses of this study rely on a combination of dependent variables that are continuous, ordinal, and binary, we tested our hypotheses by breaking down our analyses into multiple linear and classification models [64]. To test H1, Table 1 presents kernel-based regularized least squares (KRLS) models with trust in English- and Spanish-language journalists as dependent variables and consumption of English- and Spanish-language news media as the main independent variables (Models 1–2). Due to the lack of normality of the errors from an ordinary least squares regression model, we use KRLS as this method is designed to “solve regression and classification problems without relying on linearity or additivity assumptions” [65]. To test H2, we conducted ordered logistic regressions predicting attitudes towards state and local officials (Models 3–6) and a logistic regression predicting collective health (Models 7–8), all of which are presented in Table 2. In Models 3 and 4, source credibility in English- or Spanish-language journalists serves as the main independent variable. All the analyses were conducted using Stata/MP 16 software (Stata, College Station, TX, USA).

## 3. Results

The results of the KRLS models predicting trust in English and Spanish language journalists are presented in Table 1. The results of Model 2 show that the consumption of Spanish-language news is associated with higher levels of trust in Spanish-language journalists. In contrast, the results of Model 1 show no association between consumption of English-language news and trust in English-language journalists. These findings provide support for H1.

**Figure 1:**
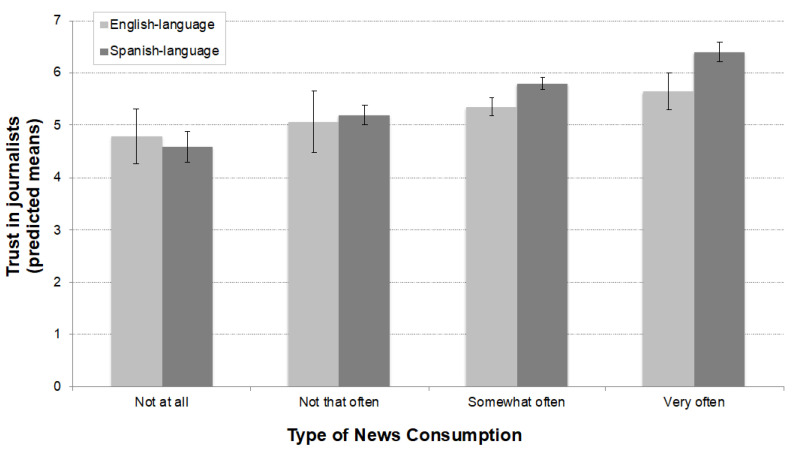
Predicted means of trust by type of news consumption.

Figure 1 provides a visual representation of Models 1 and 2. Figure 1 presents the predicted means of trust in English/Spanish-language journalists by type of news consumption (English/Spanish-language news). On one hand, the results show no statistical difference in the lowest levels of consumption of Spanish- and English-language news media (not at all, not that often) and trust in English- and Spanish-language journalists. On the other hand, there is a significant difference in trust in journalists among more avid consumers of Spanish- and English-language news. Figure 1 shows that Latinos who consume Spanish-language news media “somewhat often” and “very often” are statistically more likely to report higher levels of trust in Spanish-language journalists than Latino consumers of English-language news media and the trust they have in English-language journalists. These results provide additional support for H1.

**Figure 2:**
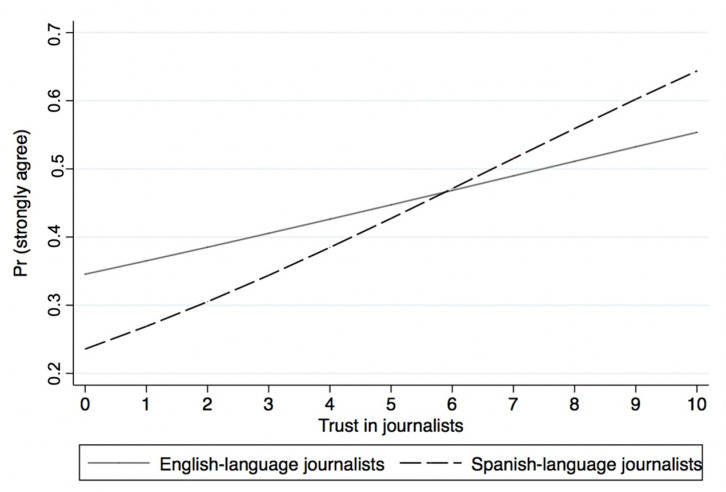
Predicted probabilities of reporting “strongly agree” on state and local officials providing clear information about what to do to stop the spread of COVID-19.

Table 2 presents the results of the ordered logistic regressions predicting attitudes towards state and local officials (Models 3–6) and logistic regressions predicting collective health (Models 7–8). The results of Models 3 and 4 show that trust in both English- and Spanish-language journalists is associated with more positive evaluations of the information provided by state and local officials about COVID-19. Figure 2 presents the predicted probabilities of reporting “strongly agree” on state and local officials providing clear information about stopping the spread of COVID-19. On one hand, Figure 2 shows that the likelihood of reporting “strongly agree” increased by 18 percentage points in the 0–10 scale on the source credibility of English-language journalists. On the other hand, the likelihood of reporting “strongly agree” increased by over 40 percentage points in the 0–10 scale on the source credibility of Spanish-language journalists. Hence, the results of Figure 2 suggest that trust in Spanish-language journalists has a larger effect on the evaluation of state and local officials’ responses to COVID-19 compared to the trust in English-language journalists. The results provide support for H2.

**Figure 3:**
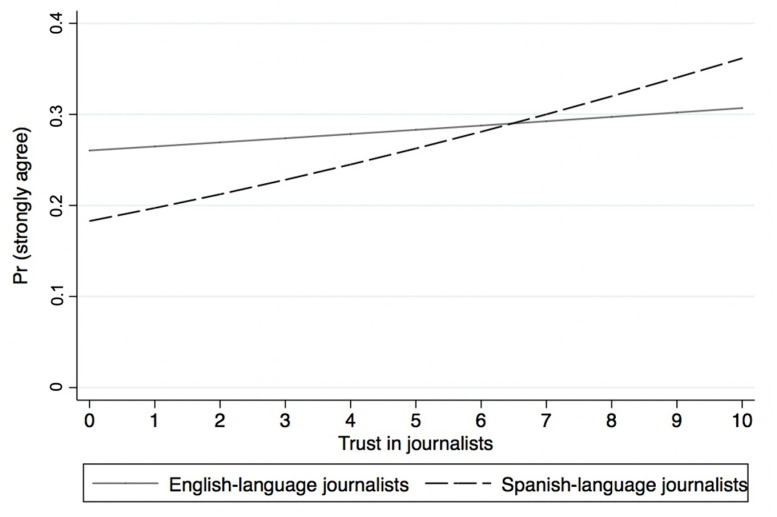
Predicted probabilities of reporting “strongly agree” on officials providing clear information on the location of testing sites.

Models 5 and 6 from Table 2 present the results of the ordered logistic regression analyses, with information from officials on the location of the nearest testing site as the main dependent variable. The results show that while there is no association between trust in English-language journalists and the perception that local officials have made it clear where the nearest testing site is located, there is a marginal relationship between the dependent variable and trust in Spanish-language journalists. Figure 3 depicts the predicted probabilities of reporting “strongly agree” on officials providing clear information on the location of testing sites. Providing additional evidence to the findings of Models 5 and 6, Figure 3 shows that while trust in Spanish-language journalists is positively and significantly associated with a positive evaluation of officials’ responses to COVID-19, the association between trust in English-language journalists and the dependent variable is null. These results provide additional support for H2.

**Figure 4:**
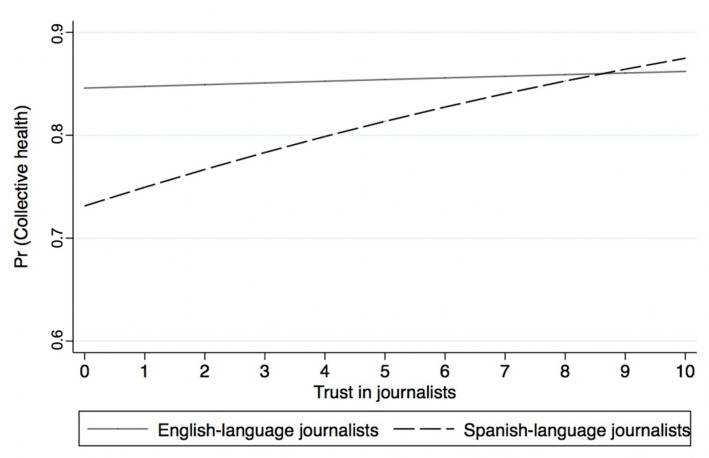
Predicted probability of supporting efforts to promote collective health by source credibility.

Models 7 and 8 from Table 2 present the results of the logistic regressions predicting collective health by source credibility. Similar to Models 5 and 6, the results show that while trust in Spanish-language journalists is statistically associated with reporting collective health, trust in English-language journalists is not. Figure 4 presents the predicted probability of supporting efforts to promote collective health by source credibility. The results show that trust in English-language journalists is not associated with reporting collective health. However, trust in Spanish-language journalists is significantly associated with a greater likelihood of supporting efforts that promote collective health. In other words, the more trust Latinos have in Spanish-language journalists, the more likely they are to believe in collective health. These results provide additional support for H2, highlighting the importance of source credibility and alternatives to mainstream media among US Latinos.

## 4. Discussion

The COVID-19 pandemic has impacted the lives of millions of Latinos in innumerable ways. Latinos have historically experienced health inequalities, which have contributed to adverse health effects during the COVID-19 pandemic [13,55,66]. Therefore, consuming trusted information is essential for Latinos’ health. The extant literature around information during infectious disease outbreaks stresses the importance of access to trustworthy, credible, and factual information to produce desirable public health outcomes [4]. Previous studies have found that shared backgrounds can impact the viewers’ perception of source credibility, particularly among racial and ethnic minorities. However, the role of ethnic and non-traditional media during infectious disease outbreaks has been understudied [35,36,37]. It is imperative to understand the relationship between Spanish-language news consumption and the trust and credibility that these sources have among Latinos during the COVID-19 pandemic.

In this study, we examined the extent to which the consumption of Spanish-language news media is associated with greater trust in Spanish-language journalists to provide accurate information related to infectious disease outbreaks and the extent to which source credibility impacts the attitude formation of Latinos during a public health crisis. These questions are valuable in both theory and practice. Latinos comprise a large percentage of the US population, and, in many ways, Spanish-language media influences their attitudes and perceptions of current events [17,67]; this is also true during the COVID-19 outbreak, as this study reveals. However, there are limitations related to the nature of the data and the ongoing challenges of the COVID-19 pandemic, which future scholarship shall address. Our analysis relies on cross-sectional data that is focused on Latinos and Latinas in the United States; this issue limits our capacity to make educated generalizations across racial and ethnic minority groups over time. Additionally, the data were collected during the first pandemic peak in the United States, a point in time that might have been socially and politically distinct to other phases of the COVID-19 pandemic as well as other pandemics and public health emergencies.

## 5. Conclusions

Given the volatile nature of rapidly changing information during the pandemic, journalists must garner the trust of their audience to spread prevention strategies and properly convey new policies and directives by public officials. Using data collected from one of the first and most comprehensive surveys of Latinos on impact and reactions to COVID-19, this study finds that the consumption of Spanish-language news media is strongly associated with source credibility among Latinos, whereas the consumption of English-language news media is not. We also find that source credibility matters for Latinos during the current public health emergency as trust in Spanish-language journalists is associated with a greater change in assessments of state and local officials providing adequate information about COVID-19. In addition, we find that while trust in Spanish-language journalists is a predictor of believing in collective health, trust in English-language journalists is not. This study has important implications as it illuminates the necessity for Spanish-language news media to provide quality information to US Latinos, who have been disproportionately impacted by COVID-19, and highlights the importance of shared backgrounds on source credibility among linguistically diverse groups. Future studies should explore the additional implications of ethnic media consumption and source credibility among underrepresented groups during public health crises.

**Figure 5:**
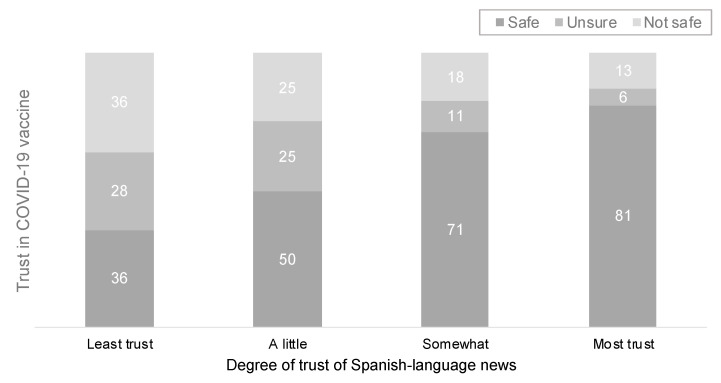
Covid-19 vaccine trust by trust in Spanish-language news among Latino adults (June 2021). Source: American COVID-19 Vaccine Poll. Notes: results presented in percentages from the question “How safe do you think the COVID-19 vaccine will be for you?”.

**Figure 6:**
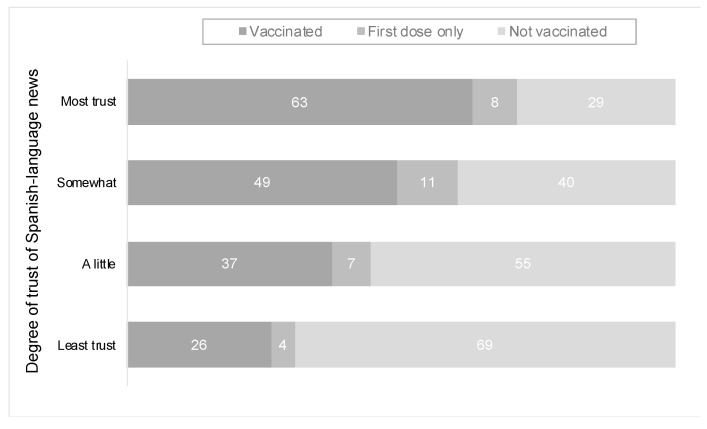
Covid-19 vaccination rates among Latino adults by trust in Spanish-language news (June 2021). Source: American COVID-19 Vaccine Poll. Notes: results presented in percentages from the question “About the COVID-19 vaccine, have you received both the first and second dose of a two-dose COVID-19 vaccine [vaccinated], received only the first dose of a two-dose COVID-19 vaccine [first dose only], received one dose of a COVID-19 vaccine that only requires one shot [vaccinated], or not had any COVID-19 vaccine [not vaccinated]”.

The implications of our findings are becoming more apparent as data on vaccination uptake and hesitancy become more available. We draw from the American COVID-19 Vaccine Poll implemented by the African American Research Collaborative, which has a large Latino sample (*n* = 2944) [68,69]. This survey, which has one of the largest Latino and AAPI samples to date, focused on vaccination status, finds that vaccination uptake among these two communities is higher for non-English speaking respondents (71% for non-English/55% English). Consistent with our findings, the American COVID-19 Vaccine Poll and other studies have revealed that trust in Spanish-language news among Latinos is associated with not only more positive assessments of the COVID-19 vaccine but also higher vaccination rates, as Figure 5 and Figure 6 show [69]. As we have argued in this paper, Spanish-language news has the capacity to build an extra layer of trust with Spanish-speaking Latinos, and, when it comes to public health practices, this relationship can deliver trustworthy facts and information about the COVID-19 vaccine and has the potential to save lives [4,7,50,53].

## Figures and Tables

**Table 1:** KRLS models predicting source credibility in English- and Spanish-language journalists.

	(1) Trust in English-Language Journalists		(2) Trust in Spanish-Language Journalists	
VARIABLES	Coefficient	SE	Coefficient	SE
English-language news consumption	0.068	0.084		
Spanish-language news consumption			0.389 ***	0.084
COVID-19 news consumption	0.305 ***	0.071	0.552 ***	0.083
Age	0.223 ***	0.069	0.093	0.084
Education	0.191 ***	0.049	0.109 **	0.051
Female	−0.525 **	0.232	0.097	0.259
US-born	0.011	0.242	0.022	0.276
SL survey	0.097	0.244	0.303	0.275
Partisanship (Democrat as base)				
Republican	−0.511 **	0.240	−0.566 **	0.273
Independent/Other	−0.746 ***	0.237	−0.403	0.262
Cuban	0.230	0.318	−0.255	0.381
Public risk perception	0.192	0.233	−0.391	0.261
Observations	542		541	

*** *p* < 0.01, ** *p* < 0.05.

**Table 2:** Models on attitudes towards information provided by state and local officials and collective health.

	What to do to Stop the Spread of COVID-19				Location of Nearest Testing Site				Collective Health			
	Model 3		Model 4		Model 5		Model 6		Model 7		Model 8	
VARIABLES	Coefficient	S.E.	Coefficient	S.E.	Coefficient	S.E.	Coefficient	S.E.	Coefficient	S.E.	Coefficient	S.E.
Trust in EL journalist	0.085 *	0.044			0.023	0.045			0.013	0.045		
Trust in SL journalist			0.177 ***	0.055			0.093 *	0.048			0.094 **	0.045
COVID-19 news consumption	0.264 **	0.133	0.567 ***	0.130	0.292 **	0.115	0.403 ***	0.110	0.391 ***	0.122	0.011	0.116
Age	0.246 **	0.124	0.170	0.132	−0.097	0.136	−0.024	0.143	0.297 **	0.135	0.125	0.133
Education	−0.080	0.087	0.030	0.090	0.040	0.091	−0.025	0.092	0.037	0.090	0.168 *	0.087
Female	0.040	0.262	0.312	0.276	−0.494 *	0.254	0.124	0.268	0.424	0.260	0.341	0.249
US-born	0.193	0.362	0.046	0.358	0.081	0.327	0.273	0.355	−0.501	0.347	0.123	0.319
SL survey	0.087	0.364	−0.762 **	0.341	0.357	0.310	0.103	0.350	−0.729 **	0.337	0.229	0.351
Partisanship	0.147	0.178	−0.042	0.187	0.485 ***	0.181	0.488 ***	0.155	−0.591 ***	0.168	−0.541 ***	0.161
Cuban	0.630	0.511	0.734	0.631	0.221	0.544	0.472	0.392	1.368 **	0.559	0.083	0.432
Public risk perception	−0.496 *	0.274	−0.247	0.299	−0.020	0.249	−0.089	0.265	0.447 *	0.259	0.553 **	0.271
Constant cut1	−1.142 *	0.692	0.056	0.904	0.565	0.756	1.227 *	0.736				
Constant cut2	0.082	0.718	1.403 *	0.845	1.587 **	0.754	2.461 ***	0.765				
Constant cut3	2.272 ***	0.750	3.771 ***	0.897	2.915 ***	0.783	4.179 ***	0.811				
Constant									0.400	0.789	0.511	0.705
Observations	270		264		270		263		542		541	

*** *p* < 0.01, ** *p* < 0.05, * *p* < 0.1. Note: S.E. stands for standard errors.

## Data Availability

The data that supports the findings of this study belongs to the African American Research Collaborative and may be available upon request from the organization. The complete instrument and survey results are available at AfricanAmericanresearch.us.

## Appendix A. Variable Construction and Summary Statistics

Trust in EL/SL journalists: On a scale of 0 to 10, where 0 means “do not trust at all” and 10 means “completely trust”, how much do you trust each of the following to provide accurate information and helpful advice related to the coronavirus outbreak? [randomize and split] English-language news journalists; Spanish-language news journalists

Stopping the spread of COVID-19: How much do you agree or disagree with the following statements? [randomize and split] “State or local officials have made it clear what I should be doing right now to stop the spread of coronavirus.” (1) Strongly disagree, (2) somewhat disagree, (3) somewhat agree, (4) strongly agree.

Location of nearest testing site: How much do you agree or disagree with the following statements? [randomize and split] “My local officials have made it clear where the nearest testing site is located, where I can get tested for coronavirus.” (1) Strongly disagree, (2) somewhat disagree, (3) somewhat agree, (4) strongly agree.

Collective health: Which statement do you agree with most? [randomize] (0) America needs to get back to work now. This has gone on too long, people are losing jobs and the economy is falling apart. It is a sad fact that some people will get sick and die when we all go back to our normal lives, but Americans need to get back to work so that the economy recovers and people keep their jobs. (1) We are all in this together and need to do whatever we can to prevent the spread of coronavirus. Even if it means more weeks of staying at home, missing work and/or school—that is a small price to pay in order to save millions of Americans from serious illness or death due to coronavirus.

English-language news consumption: When you look for news and information on TV, radio, social media, or other websites, how often do you access information from English-language outlets? (1) Not at all, (2) not that often, (3) somewhat often, (4) very often.

Spanish-language news consumption: When you look for news and information on TV, radio, social media, or other websites, how often do you access information from Spanish-language outlets? (1) Not at all, (2) not that often, (3) somewhat often, (4) very often.

COVID-19 news: How closely have you been following the news about coronavirus, also called COVID-19, an infectious virus that is affecting the US and other countries around the world? (1) Following very closely, (2) following somewhat closely, (3) not following that closely, (4) not really following at all.

Age: (1) 18–29, (2) 30–39, (3) 40–64, (4) 65 and above.

Education: What is the highest level of education you have completed? (1) Less than high school, (2) high school graduate, (3) some college, but did not graduate, (4) 4 year degree/bachelor’s degree, (5) post-graduate degree.

Female: Do you identify as (1) female or (0) male?

US-born: Were you born in (1) the United States, (0) the island of Puerto Rico, or (0) somewhere else?

Spanish-language survey [codes whether the survey was conducted in English or Spanish]: (1) Spanish, (0) English.

Party identification: Generally speaking, do you think of your political party affiliation as (1) Democrat, (2) Independent or other, or (3) Republican.

Cuban: [Hispanics/Latinos] have their roots in many different parts of Latin America. To what country or place in Latin America do you or your family trace your ancestry? (1) Cuba, (0) another Latin American country.

Public risk perception: Which of the following do you agree with most? (1) My city or county has a lot of coronavirus cases, but it is under control and not a direct threat to me or the area where I live. (2) My city or county has a serious problem with coronavirus, and many people in my community are at risk of getting sick from it, including me. (3) My city or county does not really have a problem with coronavirus. For the most part, it is not a serious threat to me or the people who live in my area.

The second and third dependent variables testing H2 are related to the effectiveness of information from state and local officials about COVID-19, including what to do to stop the spread of COVID-19 and the location of the nearest testing site; responses for these questions ranged from (1) “strongly disagree” to (4) “strongly agree”.

**Table A1:** Descriptive Statistics.

Variable	Observations	Mean	Std. Dev.	Min	Max
Trust in EL journalist	601	5.418	3.108	0	10
Trust in SL journalist	599	5.701	3.130	0	10
Stopping the spread of COVID-19	585	3.303	0.800	1	4
Location of nearest testing site	582	2.826	1.036	1	4
Collective health	1200	0.804	0.397	0	1
English-language news consumption	1200	2.215	0.916	0	3
Spanish-language news consumption	1200	1.942	1.042	0	3
COVID-19 news consumption	1200	4.165	1.041	0	5
Age	1200	2.222	1.012	1	4
Education	1200	3.003	1.539	1	5
Female	1200	0.520	0.500	0	1
US-born	1200	0.600	0.490	0	1
SL survey	1200	0.414	0.493	0	1
Partisanship	1200	1.857	0.795	1	3
Cuban	1200	0.083	0.277	0	1
Public risk perception	1200	0.438	0.496	0	1
